# Multistate Shigellosis Outbreak and Commercially Prepared Food, United States

**DOI:** 10.3201/eid1006.030599

**Published:** 2004-06

**Authors:** Akiko C. Kimura, Kammy Johnson, Mary S. Palumbo, Jacqueline Hopkins, Janice C. Boase, Roshan Reporter, Marcia Goldoft, Karen R. Stefonek, Jeff A. Farrar, Thomas J. Van Gilder, Duc J. Vugia

**Affiliations:** * California Department of Health Services, Gardena, California, USA;; † Centers for Disease Control and Prevention, Atlanta, Georgia, USA;; ‡ California Department of Health Services, Sacramento, California, USA;; § San Diego County Health and Human Services Agency, San Diego, California, USA;; ¶ Public Health-Seattle and King County, Seattle, Washington, USA;; # Los Angeles County Department of Health Services, Los Angeles, California, USA;; ** Washington State Department of Health, Shoreline, Washington, USA;; †† Department of Human Services, Portland, Oregon, USA;; ‡‡ California Department of Health Services, Berkeley, California, USA

**Keywords:** Disease outbreaks, Shigella infections, food contamination, food handling

## Abstract

In 2000, shigellosis traced to a commercially prepared dip developed in 406 persons nationwide. An ill employee may have inadvertently contaminated processing equipment. This outbreak demonstrates the vulnerability of the food supply and how infectious organisms can rapidly disseminate through point-source contamination of a widely distributed food item.

*Shigella* infects an estimated 450,000 people annually in the United States and is usually transmitted through person-to-person spread (*1**,**2*). During January 18–21, 2000, California, Oregon, and Washington reported cases of gastroenteritis that developed in several persons after they ate a commercially prepared (brand X), five-layered bean dip; stool cultures yielded *S. sonnei*. A cohort study of an outbreak at a party implicated this dip; five of six ill attendees had eaten brand X dip compared with none of six well attendees (relative risk = 7.0; 95% confidence interval 1.1 to 42.9). On January 21, the manufacturer began a voluntary recall of the bean dip. We conducted a nationwide investigation to determine the magnitude and severity of the outbreak, confirm its source, and identify the mechanism of contamination.

## The Study

A case was defined as *S. sonnei* gastroenteritis that developed within 5 days of a person’s consuming brand X five-layered dip. Case-patient follow-up varied by health jurisdiction. In California, detailed interviews were systematically attempted with all case-patients by using a standardized questionnaire.

An environmental investigation of the brand X dip-production facility was completed. It included a review of production procedures and product distribution as well as collection of environmental samples for culture. Employees were interviewed, and stool specimens were collected. Pulsed-field gel electrophoresis (PFGE) patterns of isolates were compared by using PulseNet (*3*). Antimicrobial susceptibility patterns were obtained from clinical laboratories when possible.

We identified 406 cases in 10 states. Fourteen persons were hospitalized; no deaths were reported. Cases were primarily from the western United States: 217 (53%) from California, 132 (33%) Washington, and 31 (8%) Oregon (Table).

**Table:** Culture-confirmed *Shigella sonnei* cases associated with five-layered bean dip, by patients’ state of residence, January 2000

State	No. of cases
Alaska	1
Arizona	1
California	217
Idaho	13
Illinois	2
Minnesota	1
Oregon	31
New Mexico	2
Nevada	6
Washington	132
Total	406

Details of the California outbreak were used to characterize the larger multistate outbreak. Illness onsets ranged from January 8 through February 2, 2000 (Figure). The median incubation period was 2 days (range 1–5 days). The median age of confirmed case-patients was 35 years (range 1–79 years); 65% were female. In addition to diarrhea, the most commonly reported signs and symptoms were abdominal cramps (96%), fever (92%), vomiting (51%), and bloody diarrhea (46%). Most (93%) patients were seen by a physician; 82% were prescribed antimicrobial agents, usually a fluoroquinolone. The median duration of diarrheal illness was 7 days (range 2–21 days).

**Figure:**
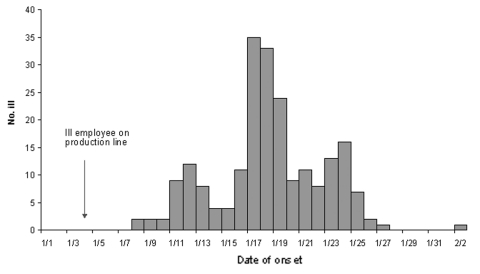
Illness onset dates of persons who ate brand X five-layered bean dip, California, January–February, 2000 (N = 217).

The dip was a refrigerated product consisting of five layers: beans, salsa, guacamole, nacho cheese, and sour cream. Each layer was prepared and placed in cold storage before manual assembly of the finished product. Preservative (sodium benzoate, 0.1%), when used, was added to individual layers. The beans were the only ingredient cooked during processing. The guacamole and salsa layers contained fresh, raw ingredients and were sold as individual products with and without preservative in addition to being used in the dip. The remaining layers were prepared solely for the dip.

The cheese layer was prepared in large batches by the same employee once or twice a week. Blocks of cheese were cut into chunks with a knife, broken into pieces by hand, and placed into a colloid mill, which sheared the mixture into a paste. When disassembled during the environmental investigation, the colloid mill had a build-up of residue on the shearing mechanism, a part reported as being difficult to clean properly. In addition, the facility had numerous violations of good manufacturing practices (*4*), including lack of standard operating procedures (*4*), inadequate refrigeration of the product, and inadequate cleaning and sanitation of processing equipment.

Product distribution was nationwide through a number of vendors but primarily through grocery chain A (17%) and warehouse store B (76%). Both facilities sold the dip without preservatives only in their West Coast stores. Brand X dip production and distribution records were incomplete; however, on the basis of recalled expiration dates, estimated production dates were December 28, 1999–January 18, 2000. All employees working on the dip production line were questioned about gastrointestinal illness just before and during the suspected production period of the contaminated dip. Only one employee reported having gastroenteritis during that period. He went home ill with diarrhea on January 3 and returned to work on January 5 (the plant was not in operation January 4). Stool cultures were not taken at the time of his illness, and he was not given antimicrobial agents; diarrhea reportedly lasted 1 day. He was an hourly employee and had no paid sick leave. Breaking the cheese up by hand and feeding it through the colloid mill were solely the responsibility of this employee.

The PFGE patterns of the outbreak isolates were either indistinguishable (pattern A) or differed by only 1–2 bands. *S. sonnei* with PFGE pattern A was isolated from an unopened container of brand X five-layered bean dip. Antibiograms of *S. sonnei* isolates from 22 case-patients were reviewed; all but one were resistant to both ampicillin and trimethoprim-sulfamethoxazole (TMP-SMZ).

Stool cultures from all employees on the dip production line were negative. Environmental and fresh produce cultures also tested negative for bacterial pathogens; however, cultures of the colloid mixer residue collected on February 2 grew 410,000 coliforms/g but were negative for *Shigella*.

## Conclusions

In January and February 2000, we identified 406 cases of drug-resistant shigellosis in 10 states in persons who had eaten brand X five-layered dip. This dip was epidemiologically implicated as the outbreak source through a cohort study and supported by these findings: 1) patients in several states whose only reported common exposure was the dip and who had PFGE-matched *S. sonnei* infections; 2) isolation of *S. sonnei* PFGE pattern A from brand X dip; and 3) outbreak termination after the product was recalled.

Numerous problems in manufacturing practices were noted at the dip-production facility, which suggests that the contamination occurred there, probably by an infected employee. Large-scale shigellosis outbreaks caused by infected food handlers are not uncommon (*5**,**6*) and are frequently attributed to poor food-handler hygiene (*7*). The employee who broke up the cheese by hand reported illness consistent with mild shigellosis during the suspected production period of the implicated product. Though culture-negative, his stool specimens were collected >3 weeks after his symptoms resolved. If this worker inadvertently contaminated the cheese, S*higella* may then have propagated in the colloid mixer, which was not cleaned regularly and was stored in a non–air-conditioned room. The wide range of illness onsets and product expiration dates suggests that contamination occurred on more than one production date, a fact that further supports this hypothesis.

The possibility that this outbreak is produce-related cannot be discounted, particularly since several shigellosis outbreaks have been due to fresh produce (*8**,**9*). PFGE pattern A was seen in a parsley-associated outbreak of *S. sonnei* in the summer of 1998 (*8*). However, despite enhanced surveillance, no illness in persons who ate brand X guacamole or salsa—the only layers containing fresh produce—as stand-alone products was reported. These products were not part of the recall.

The *S. sonnei* isolates from this outbreak were resistant to both ampicillin and TMP-SMZ. This finding has clinical implications because TMP-SMZ has been the treatment of choice for shigellosis acquired in the United States (*2*). This resistance pattern is common in developing countries, where antimicrobial use is relatively unrestricted, but it has been seen with increasing frequency in the United States (*10**,**11*). Most patients in this outbreak were treated with fluoroquinolones, to which the organism was sensitive.

Most cases occurred in western states, where the dip without preservative was distributed. The antimicrobial effects of the food preservative sodium benzoate have been well-documented (*12**,**13*). Having a preservative-containing alternative may have averted a more extensive outbreak of disease.

The evolving epidemiology of foodborne outbreaks reflects changes in the way that food is processed and distributed (*14*). The consumer can be educated to cook or wash minimally processed products such as raw meats, eggs, and fresh produce thoroughly before eating. However, in the case of a ready-to-eat product such as this dip, the responsibility to ensure safety of the product before opening rests with the growers, manufacturers, distributors, and retailers. Increasing emphasis is being placed on improving food safety through identifying and controlling potential hazards. These establishments also need to provide frequent, linguistically appropriate food safety training for all employees and remove financial disincentives for employees with gastrointestinal illnesses.

In this outbreak, a drug-resistant, virulent organism was rapidly disseminated through a commercially processed product. Although this outbreak was likely unintentional, it illustrates the vulnerability of the food supply, which is increasingly characterized by centralized production and broad distribution, and the potential for commercially produced food to be used in an act of bioterrorism. Intentional contamination of a ready-to-eat, widely distributed food product with an organism that has a low infectious dose (e.g., *Shigella*) can cause considerable illness and can be extremely costly in terms of personal, medical, and public health resources (*15*). Whether intentional or unintentional, early, open lines of communication between local and state public health departments, the Centers for Disease Control and Prevention, regulatory agencies, industry, clinicians, and consumers are critical in identifying and terminating a widely disseminated outbreak. Continued preparedness of the public health community at all levels to respond to foodborne events through protocol development and exercises designed to test their adequacy is also needed.
